# The landscape of immune checkpoint inhibitor therapy in advanced lung cancer

**DOI:** 10.1186/s12885-021-08662-2

**Published:** 2021-08-28

**Authors:** Chengdi Wang, Jingwei Li, Qiran Zhang, Jiayang Wu, Yuxuan Xiao, Lujia Song, Hanlin Gong, Yalun Li

**Affiliations:** 1 Department of Respiratory and Critical Care Medicine, West China Medical School/West China Hospital, Sichuan University, Chengdu, 610041 Sichuan Province China; 2 West China Medical School/West China Hospital, Sichuan University, Chengdu, China; 3 West China School of Public Health/West China Fourth Hospital, Sichuan University, Chengdu, China; 4 West China School/Hospital of Stomatology, Sichuan University, Chengdu, China; 5 Department of integrated Traditional Chinese and Western Medicine, West China Medical School/West China Hospital, Sichuan University, Chengdu, 610041 Sichuan Province China

**Keywords:** Immune checkpoint inhibitor, Efficacy, Non-small-cell lung cancer, Small-cell lung cancer

## Abstract

**Background:**

The advent of immune checkpoint inhibitors (ICIs) therapy has resulted in significant survival benefits in patients with non-small-cell lung cancer (NSCLC) without increasing toxicity. However, the utilisation of immunotherapy for small-cell lung cancer (SCLC) remains unclear, with a scarcity of systematic comparisons of therapeutic effects and safety of immunotherapy in these two major lung cancer subtypes. Herein, we aimed to provide a comprehensive landscape of immunotherapy and systematically review its specific efficacy and safety in advanced lung cancer, accounting for histological types.

**Methods:**

We identified studies assessing immunotherapy for lung cancer with predefined endpoints, including overall survival (OS), progression-free survival (PFS), objective response rate (ORR), and treatment-related adverse events (TRAE), from PubMed, Embase, Medline, and Cochrane library. A random-effects or fixed-effect model was adopted according to different settings.

**Results:**

Overall, 38 trials with 20,173 patients with lung cancer were included in this study. ICI therapy resulted in a significantly prolonged survival in both patients with NSCLC and SCLC when compared with chemotherapy (hazard ratio [HR] = 0.74; 95% confidence interval [CI], 0.70–0.79] and [HR = 0.82; 95% CI, 0.75–0.90], respectively). The magnitude of disease control and survival benefits appeared superior with ICI plus standard of care (SOC) when compared with SOC alone. OS and PFS advantages were observed only when immunotherapy was employed as the first-line treatment in patients with SCLC.

**Conclusion:**

ICI therapy is a promising therapeutic option in patients with NSCLC and SCLC. ICI plus SOC can be recommended as the optimal first-line treatment for patients with SCLC, and double-target ICIs combined with SOC are recommended in patients with NSCLC as both the first and subsequent lines of treatment. Additionally, non-first-line immunotherapy is not recommended in patients with SCLC.

**Supplementary Information:**

The online version contains supplementary material available at 10.1186/s12885-021-08662-2.

## Introduction

Lung cancer is the primary cause of cancer-related mortality and incidence, resulting in a significant economic burden [1]. Regarding histological types, lung cancer can be categorised into small-cell lung cancer (SCLC) and non-small-cell lung cancer (NSCLC). SCLC accounts for only 15% of lung cancers, with first-line treatment mainly restricted to chemotherapy or radiotherapy and presenting a worse prognosis than NSCLC [2]. In contrast, NSCLC constitutes approximately 85% of lung cancers and presents a relatively superior prognosis, given the rapid development of therapeutic techniques, including surgery, chemotherapy, radiotherapy, and targeted therapy [3, 4]; however, the actual 5-year overall survival (OS) of NSCLC remains poor. Standard of care (SOC) therapies include chemotherapy and radiotherapy for patients with lung cancer lacking specific therapeutic targets, whereas targeted therapy can be administered to those with corresponding mutated genes.

One main hypothesis for tumour invasion and metastasis is immune evasion, controlled by a combination of inhibitory or stimulatory receptors and corresponding ligands [5]. Among them, cytotoxic T-lymphocyte antigen-4 (CTLA-4) and programmed cell death 1 (PD-1) pathways are promising therapeutic targets, also known as immune checkpoints [6, 7]. Tumour cells can escape the immune system attack via forming immune checkpoints. Accordingly, blocking such immune checkpoints can activate the immune system and prevent tumour cell evasion. Currently, immune checkpoint inhibitors (ICIs) developed to treat malignant tumours, including lung cancer, can be classified into anti-PD-1, anti-PD-L1, and anti-CTLA-4 antibodies.

Accumulating evidence has reported that ICIs have higher efficacy than SOC in both NSCLC and SCLC, indicating their superior therapeutic potential. In patients with advanced NSCLC, anti-PD-1 monotherapy can achieve a median OS of 11.9 months, which was significantly superior to that with a SOC at 9.5 months (hazard ratio [HR]: 0.75; 95% confidence interval [CI]: 0.61–0.93). Furthermore, the incidence of treatment-related adverse events (TRAE) in the ICI group was reportedly lower than in the SOC group [8]. In patients with SCLC, anti-PD-L1 therapy as first-line treatment has demonstrated a better OS than platinum-etoposide treatment [9].

However, clinical trials evaluating the efficiency and safety of ICI therapy have mainly focused on NSCLC in recent years, neglecting any specific data analysis for SCLC. More importantly, systematic studies comparing ICI therapy among NSCLC patients with SCLC remain scarce.

A pooled analysis not restricted to patients with SCLC or NSCLC could provide valuable clinical information regarding anti-PD-1/PD-L1 and CTLA-4 treatments. In the present study, we aimed to validate whether immunotherapy could result in more manageable TRAEs and better efficacy than SOC in patients with advanced NSCLC or SCLC. Moreover, we compared the distinct benefits and risks of immunotherapy between patients with NSCLC and SCLC. We anticipate that our results could benefit the development of immunotherapy in lung cancer and offer practical solutions for routine clinical practice using immunotherapy in patients with NSCLC or SCLC.

## Methods

This systematic review and meta-analysis followed the Preferred Reporting Items for Systematic Reviews and Meta-analyses (PRISMA) guidelines [10].

### Search strategy and study selection

We performed a search for eligible randomised controlled trials (RCTs) from January 2010 to May 2021 in Medline, PubMed, Embase, and the Cochrane Central Register of Controlled Trials, using the following key words: ICIs (PD-1, PD-L1, or CTLA-4), specific ICI drug names (toripalimab, sintilimab, camrelizumab, tilelizumab, nivolumab, pembrolizumab, atezolizumab, durvalumab, avelumab, ipilimumab, and tremelimumab), and lung cancer. For further identifying unpublished studies, we retrieved abstracts from the American Society of Clinical Oncology, the European Society of Medical Oncology, the American Association for Cancer Research, and the World Conference on Lung Cancer. (Table S1).

Exclusion and inclusion criteria were predefined. Eligible RCTs were required to meet the following criteria: (a) population: diagnosed with lung cancer (NSCLC or SCLC) pathologically; (b) intervention: treatment with PD-1/PD-L1 or CTLA-4 inhibitors (toripalimab, sintilimab, camrelizumab, tislelizumab, nivolumab, pembrolizumab, atezolizumab, durvalumab, avelumab, ipilimumab and tremelimumab); (c) control: treated with chemotherapy or radiotherapy; (d) type of study: phase II and III clinical trials. Exclusion criteria were as follows: (a) the study was not a randomised controlled trial; (b) data regarding PFS/OS measured by HRs, objective response rate (ORR), or TRAEs was unavailable; (c) duplicate articles.

### Data extraction and quality assessment

For all included trials, we extracted the name of the trial, year of publication, trial phase, line of treatment, age and number of patients, OS/PFS/ORR, and TRAEs of grade ≥ 3 and any grade. We adopted the Cochrane Risk of Bias Tool, consisting of allocation concealment, random sequence generation, blinding of outcome assessment, blinding protocol, selective reporting, and incomplete outcome data, to methodologically assess the quality of the enrolled RCTs [11]. The items adjudged as “low risk” were regarded as applicable. Two authors independently performed data extraction and quality assessment. Discrepancies were resolved by reaching a consensus.

### Statistical analysis

Heterogeneity was identified by the Q test and quantified using the I^2^ and Q statistics [12]. If I^2^ was more than 50%, the random effect model was applied; otherwise, the fixed-effect model was selected [13]. The primary outcomes in the present study were OS and PFS, measured as HRs, 95% CIs, and *p*-values. ORR, grade ≥ 3 TRAEs, and Grade 1–5 TRAEs were presented as risk ratios (RRs). The Q test was used to detect heterogeneity between the subgroups and assess differences between histological types. Prespecified subgroup analyses were performed to evaluate the potential association between individual or methodological factors and immunotherapy efficacy in each histological type of lung cancer. Eggerʼs and Beggʼs tests were used to assess the publication bias for included RCTs. Stata 16.0 software (Stata Corp, College Station, TX) was used to perform all analyses. Statistical significance was set at *p* < 0.05.

## Result

### Literature search results

**Fig. 1:**
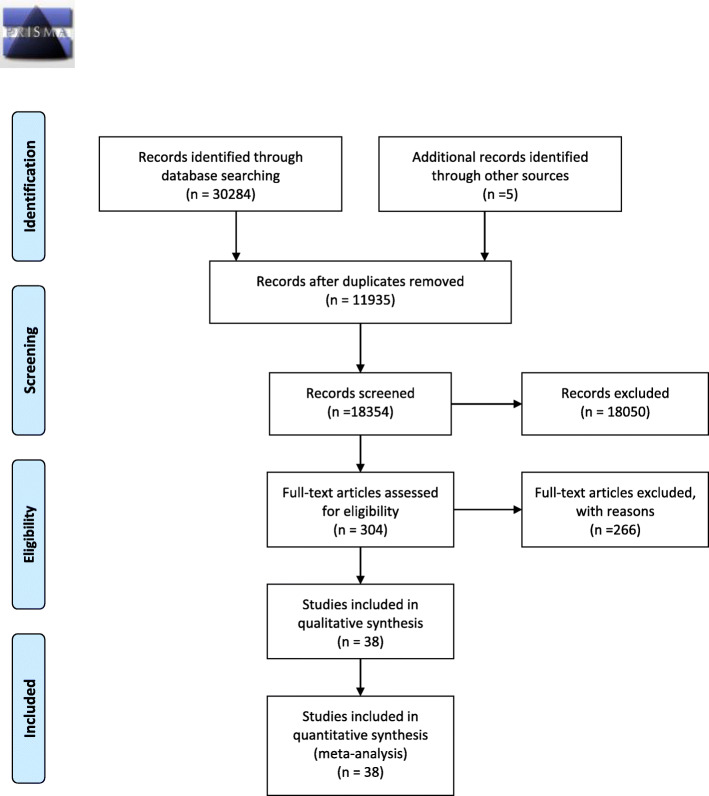
Flowchart diagram of selected randomized controlled trials included

The initial literature search identified 30,284 related studies (Fig. 1). In total, 38 RCTs, including 41 studies with 20,173 patients with lung cancer, were included for quantitative analyses [6–8, 14–48]. Eight studies explored the efficacy of ICI versus SOC in patients with SCLC (three studies on ipilimumab, two on atezolizumab, one on nivolumab, one about durvalumab, and one assessing tremelimumab plus durvalumab). The remaining 33 studies were performed efficiency and safety comparisons between ICIs and SOC in patients with NSCLC.

### Characteristics of identified trials

The main characteristics of the 38 trials are listed in Table 1. All included trials were performed in patients with relapsed or extensive SCLC and advanced NSCLC. In total, 20,173 patients were included, of which 17,250 (85.5%) were diagnosed with NSCLC and 2923 (14.5%) with SCLC. Regarding age, most patients were ≥ 70 years old. All eligible trials were phase II or III studies, with 31 phase III trials, 6 phase II, and 1 phase II/III. Among these trials, 22 employed ICIs as first-line therapy, and the remaining trials were in a non-first-line setting. Overall, all studies, except for 17 (44.7%), confirmed improvements in OS in patients receiving immunotherapy when compared with those receiving SOC. Except for PEMBRO-RT and IFCT-1603, all trials reported total TRAEs in patients. Furthermore, several RCTs were uniquely designed, necessitating further explanation. KEYNOTE-010 evaluated the efficiency of different pembrolizumab doses (2 mg/kg and 10 mg/kg), accordingly divided into KEYNOTE-010, a and KEYNOTE-010, b. OAK established two different cohorts, ITT850 and ITT1225, both of which were treated as independent studies. Likewise, ARCTIC and CASPIAN were considered independent studies. CA184–041 was a phase II study focusing on different medication orders, which was considered four studies based on histological type and order of medication. Trials were generated through a random sequence and at low risk of selection bias, presenting good quality (Table S2). The reduced selection bias was attributed to low attrition and thorough reporting without missing cases. The funnel plot (Fig. S1), as well as Eggerʼs and Beggʼs tests, all indicated no sign of publication bias.

**Table 1:** Clinical characteristics and outcomes of the included randomized controlled trials

Trials	Trial phase	Line of treatment	Intervention (No.)	Control (No.)	Age, Median (Range)	Efficiency			TRAEs	
						OS (95% CI)	PFS (95% CI)	ORR	Grade 3–5	Grade 1–5
**NSCLC**										
KEYNOTE-407, 2018	III	1	PEM plus PBC (278)	PBC plus placebo (281)	Int:65 (29–87) Con:65 (36–88)	0.64 (0.49–0.85)	0.56 (0.45–0.70)	161/278 108/281	194/278 191/280	273/278 274/280
KEYNOTE-021, 2016	II	1	PEM plus PBC (60)	PBC (63)	Int:62.5 (54–70) Con:63.2 (58–70)	0.56 (0.32–0.95)	0.53 (0.33–0.86)	33/60 18/63	24/59 17/62	55/59 57/62
OAK ITT850 2017, 2019	III	> 1	ATE (425)	DOC (425)	Int:63.5 (33–77) Con:58.5 (34–79)	0.75 (0.64–0.88)	0·95 (0·82–1·10)	58/425 57/425	90/609 248/578	390/609 496/578
CheckMate 026 2017	III	1	NIV (271)	PBC (270)	Int:63 (32–89) Con:65 (29–87)	1.08 (0.87–1.34)	1.19 (0.97–1.46)	55/211 71/212	47/267 133/263	190/267 243/263
OAK ITT1225 2018	III	> 1	ATE (613)	DOC (612)	Int:63 (25–84) Con:64 (34–85)	0.80 (0.70–0.92)	0.96 (0.85–1.08)	84/613 72/612	243/609 322/578	574/609 557/578
JAVELIN Lung 200, 2018	III	> 1	Avelumab (396)	DOC (396)	Int:64 (58–69) Con:63 (57–69)	0·90 (0·75–1·08)	1·16 (0·97–1·40)	59/396 44/396	39/393 180/365	251/393 254/365
KEYNOTE-189, 2018	III	1	PEM plus PBC (410)	PBC plus placebo (206)	Int:65 (34–84) Con:63 (34–84)	0.49 (0.38–0.64)	0.52 (0.43–0.64)	195/410 39/206	272/405 133/202	404/405 200/202
KEYNOTE-042, 2019	III	1	PEM (637)	PBC (637)	Int:63 (57–69) Con:63 (57–69)	0.81 (0.71–0.93)	1.07 (0.94–1.21)	174/637 169/637	113/636 252/615	399/636 553/615
KEYNOTE-010, a, 2016	II/III	> 1	PEM (344)	DOC (172)	Int:63 (56–69) Con:62 (56–69)	0.71 (0.58–0.88)	0.88 (0.74–1.05)	62/344 16/172	43 /339 55/155	215/339 126/155
KEYNOTE-010, b, 2016	II/III	> 1	PEM (346)	DOC (171)	Int:63 (56–69) Con:62 (56–69)	0.61 (0.49–0.75)	0.79 (0.66–0.94)	64/346 16/171	55/343 54/154	226/343 125/154
POPLAR, 2016	II	> 1	ATE (144)	DOC (143)	Int:62 (42–82) Con:62 (36–84)	0.73 (0.53–0.99)	0.94 (0.72–1.23)	21/144 21/143	57/142 71/135	95/142 119/135
PACIFIC 2017, 2018	III	> 1	DUR (476)	PBC plus Placebo (237)	Int:64 (31–84) Con:64 (23–90)	0.68 (0.47–0.997)	0.52 (0.42–0.65)	126/443 34/123	142/475 61/234	460/475 222/234
KEYNOTE- 024, 2016, 2019	III	1	PEM (154)	PBC (151)	Int:64.5 (33–90) Con:66.0 (38–85)	0.63 (0.47–0.86)	0.50 (0.37–0.68)	69/154 42/151	48/154 80/150	118/154 135/150
CheckMate 017 2015	III	> 1	NIV (135)	DOC (137)	Int:62 (39–85) Con:64 (42–84)	0.59 (0.44–0.79)	0.62 (0.47–0.81)	27/135 12/137	9/131 71/129	76/131 111/129
IMpower110 2020	III	1	ATE (277)	PBC (277)	Int:64 (30–81) Con:65 (30–87)	0.83 (0.65–1.07)	0.77 (0.63–0.94)	81/277 88/277	97/286 149/263	258/286 249/263
CheckMate 057 2015	III	> 1	NIV (292)	DOC (290)	Int:61 (37–84) Con:64 (21–85)	0.73 (0.59–0.89)	0.92 (0.77–1.11)	56/292 36/290	30/287 144/268	199/287 236/268
IMpower150 2018	III	1	ATE plus PBC (400)	PBC (400)	Int:63 (31–89) Con:63 (31–90)	0.78 (0.64–0.96)	0.61 (0.52–0.72)	224/353 159/331	230/393 197/394	371/393 376/394
CheckMate 078 2020	III	> 1	NIV (338)	DOC (166)	Int:60 (27 to 78) Con:60 (38 to 78)	0.75 (0.61–0.93)	: 0.79 (0.65–0.98)	59/338 7/166	41/337 74/156	219/337 131/156
IMpower130 2019	III	1	ATE plus PBC (483)	PBC (240)	Int:64 (18–86) Con:65 (38–85)	0·80 (0·65–0·99)	0.65 (0·54–0·77)	220/447 72/226	354/473 141/232	455/473 215/232
ARCTIC, a, 2020	III	> 1	DUR (62)	Erlotinib, gemcitabine, or vinorelbine) (64)	Int:63.5 (35–79) Con:62.0 (41–81)	0.63 (0.42–0.93)	0.71 (0.49–1.04)	22/62 8/64	25/62 41/63	60/62 63/63
ARCTIC, b 2020	III	> 1	DUR plus TRE (174)	Erlotinib, gemcitabine, or vinorelbine) (118)	Int:62.5 (26–81) Con:65 (42–83)	0.80 (0.61–1.05)	0.77 (0.59–1.01)	26/174 8/118	74/173 57/110	160/173 105/110
CameL 2020	III	1	CAM plus PBC (205)	PBC (207)	Int:59 (54–64) Con:61 (53–65)	0.73 (0.53–1.02)	0.60 (0.45–0.79)	124/205 80/207	78/205 63/207	146/205 132/207
CheckMate 227 2019	III	1	NIV plus IPI (583)	PBC (583)	Int:64 (26–87) Con:64 (29–87)	0.73 (0.64–0.84)	0.79 (0.69–0.91)	199/583 162/583	189/576 205/570	442/576 467/570
CheckMate 9LA 2021	III	1	NIV plus IPI plus PBC (361)	PBC (358)	Int:65 (59–70) Con:65 (58–70)	0·69 (0·55–0·80)	0·68 (0·57–0·82)	138/361 89/358	168/358 132/349	327/358 303/349
CA184–041, a 2012	II	1	Concurrent IPI plus PBC (70)	PBC (33)	Int:59 (36–82) Con:62 (36–82)	0.99 (0.67–1.46)	0.88 (0.61–1.27)	15/70 6/33	40/71 13/32	52/71 23/32
CA184–041, b 2012	II	1	Phased IPI plus PBC (68)	PBC (33)	Int:61 (36–82) Con:62 (36–88)	0.87 (0.59–1.28)	0.69 (0.48–1.00)	22/68 6/33	36/67 13/33	49/67 23/33
CA184–104 2017	III	1	IPI plus PBC (388)	PBC plus placebo (361)	Int:64 (28–84) Con:64 (28–85)	0.91 (0.77–1.07)	0.87 (0.75–1.01)	171/388 170/361	205/388 129/361	344/388 292/361
IMpower132 2020	III	1	ATE plus PBC (292)	PBC (286)	Int:64 (31–85) Con:63 (33–83)	0.86 (0.71–1.06)	0.60 (0.49–0.72)	137/292 92/286	208/291 166/274	287/291 266/274
PEMBRO-RT 2019	II	> 1	PEM plus Radiotherapy (36)	Radiotherapy (40)	Int:62 (35–78) Con:62 (38–78)	0.66 (0.37–1.18)	0.71 (0.42–1.18)	13/36 7/40	12/35 6/37	NA
IMpower131 2020	III	1	ATE plus PBC (343)	PBC (340)	Int:65 (23–83) Con:65 (38–86)	0.88 (0.73–1.05)	0.71 (0.60–0.85)	170/343 139/340	231/334 195/334	316/334 303/334
EMPOWER-Lung 1 2021	III	1	CEM (283)	PBC (280)	Int:63 (58–69) Con:64 (58–70)	0.57 (0.42–0.77)	0.54 (0.43–0.68)	111/283 57/280	50/355 134/342	204/355 303/342
RATIONALE 307, a 2021	III	1	TIS plus PBC (120)	PBC (61)	Int:60 (41–74) Con:62 (34–74)	\	0.52 (0.37–0.74)	87/120 30/61	103/120 47/59	119/120 59/59
RATIONALE 307, b 2021	III	1	TIS plus PBC (119)	PBC (60)	Int:63 (38–74) Con:62 (34–74)	\	0.48 (0.34–0.68)	89/119 30/60	99/118 47/58	117/118 58/58
**SCLC**										
CASPIAN, a 2021	III	1	TRE plus DUR plus PBC (268)	PBC (269)	Int:63 (58–68) Con:63 (57–68)	0·82 (0·68–1·00)	0·84 (0·70–1·01)	156/267 78/134	196/266 86/133	264/266 129/133
CASPIAN, b 2021	III	1	DUR plus PBC (268)	PBC (269)	Int:62 (58–68) Con:63 (57–68)	0.75 (0.62–0.91)	0·80 (0·66–0·96)	182/268 78/135	171/265 87/133	260/265 129/133
IFCT-1603 2019	II	> 1	ATE (49)	PBC (24)	Int:65.9 (51.1–85.5) Con:63.5 (51.8–81.0)	0.84 (0.45–1.58)	2.26 (1.30–3.39)	1/43 2/20	2/48 18/24	NA
IMpower133 2018	III	1	ATE plus PBC (201)	PBC plus placebo (202)	Int:64 (28–90) Con:64 (26–87)	0.70 (0.54–0.91)	0.77 (0.62–0.96)	121/201 130/202	115/198 113/196	188/198 181/198
CA184–041, a 2013	II	1	Concurrent IPI plus PBC (43)	PBC plus placebo (23)	Int:57 (44–80) Con:58 (42–82)	0.95 (0.59–1.54)	0.93 (0.59–1.48)	14/43 11/23	19/42 10/22	29/42 18/22
CA184–041, b 2013	II	1	Phased IPI plus PBC (42)	Placebo plus PBC (22)	Int:59 (43–80) Con:58 (42–82)	0.75 (0.46–1.23)	0.93 (0.59–1.45)	24/42 11/22	22/42 9/22	33/42 18/22
CA184–156, 2016	III	1	IPI plus PBC (478)	Placebo plus PBC (476)	Int:62 (39–85) Con:63 (36–81)	0.94 (0.81–1.09)	0.85 (0.75–0.97)	297/478 296/476	231/478 214/476	391/478 361/478
CheckMate 331 2021	III	> 1	NIV (284)	PBC (285)	Int:62 (37–85) Con:61 (34–82)	0.86 (0.72–1.04)	1.41 (1.18–1.69)	39/284 47/285	39/282 194/265	156/282 239/265

*Abbreviations*: *ATE* atezolizumab, *AVE* avelumab, *DOC* docetaxel, *TRAE* treatment-related adverse event, *IPI* ipilimumab, *NIV* nivolumab, *DUR* durvalumab, *TRE* tremelimumab, *CAM* camrelizumab, *CEM* Cemiplimab, *TIS* Tislelizumab, *ORR* objective response rate, *OS* overall survival, *PBC* platinum-based chemotherapy, *PEM* pembrolizumab, *PFS* progression-free survival

### Efficiency

**Fig. 2:**
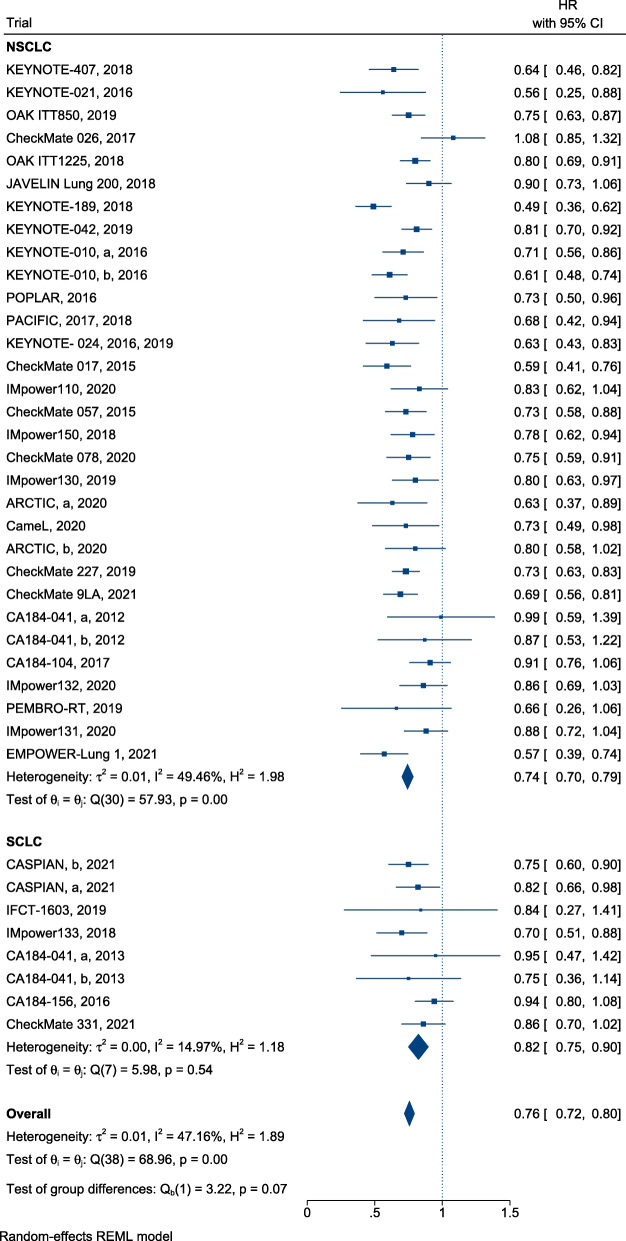
Forest plots of HRs comparing overall survival of immunotherapy between NSCLC and SCLC

**Fig. 3:**
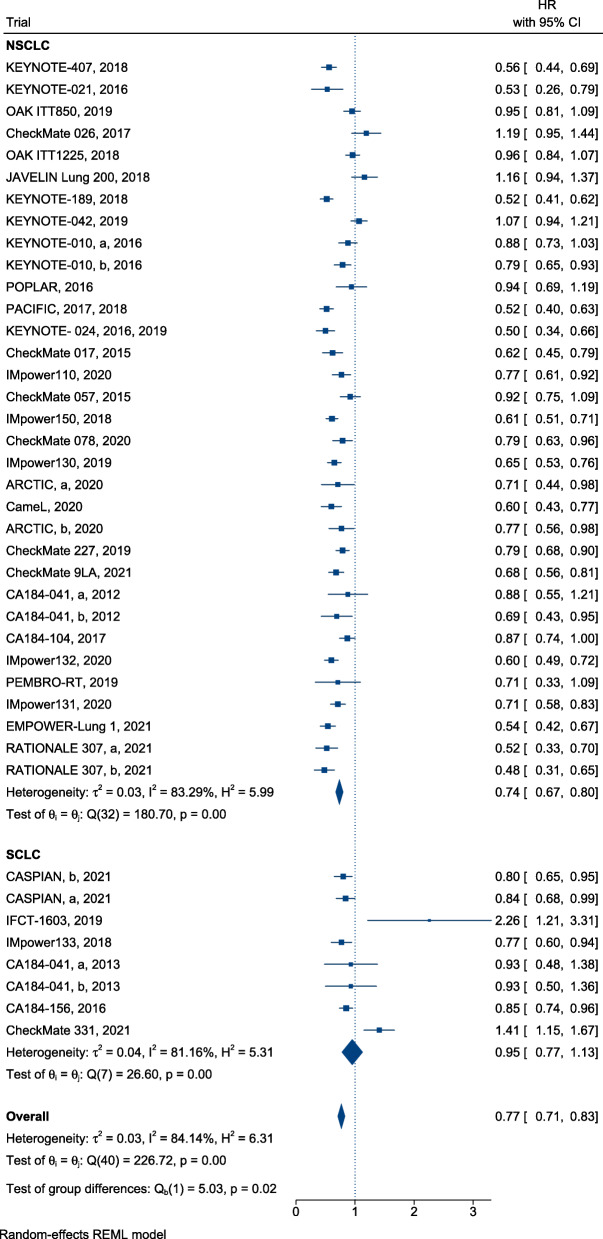
Forest plots of HRs comparing progression-free survival of immunotherapy between NSCLC and SCLC

**Fig. 4:**
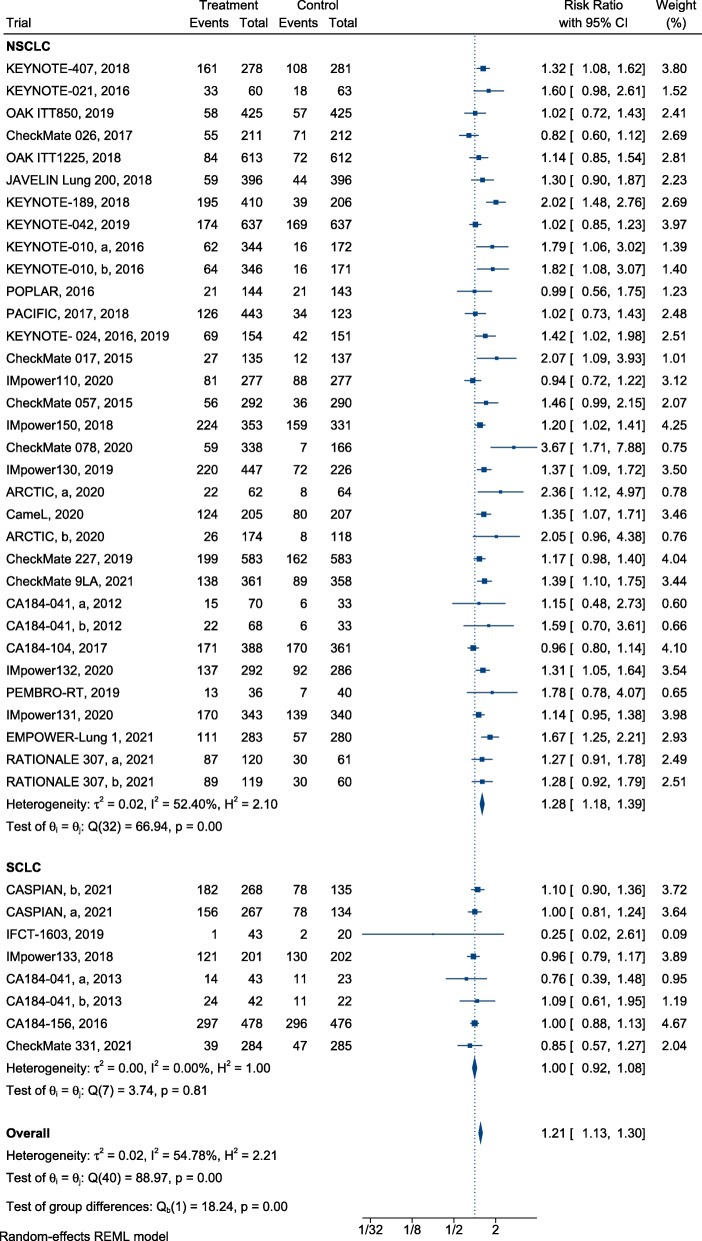
Forest plots of RRs comparing overall response rate of immunotherapy between NSCLC and SCLC

In summary, ICI treatment presented a significant advantage over SOC, with a reduction in mortality (HR, 0.76; 95% CI, 0.72–0.80) (Fig. 2) and successful control of disease progression in patients with lung cancer (HR, 0.77; 95% confidence interval [CI], 0.71–0.83) (Fig. 3). Furthermore, immunotherapy yielded superior efficacy in terms of objective response in patients with lung cancer when compared with chemotherapy or radiotherapy (RR, 1.21; 95% CI, 1.13–1.30; Fig. 4). Regarding different histological types, greater improvements in PFS following ICI therapy were observed in patients with NSCLC than in patients with SCLC ([HR = 0.74; 95% CI, 0.67–0.80] and [HR = 0.95; 95% CI, 0.77–1.13]; difference *p* = 0.02; Fig. 3), similar findings were documented in terms of ORR ([RR = 1.28; 95% CI, 1.18–1.39] and [RR = 1.00; 95% CI, 0.92–1.08]; difference *p* < 0.01; Fig. 4). In contrast, equivalent OS benefits from ICI therapy were observed in both patients with NSCLC and SCLC ([HR = 0.74; 95% CI, 0.70–0.79] and [HR = 0.82; 95% CI, 0.75–0.90]; difference *p* = 0.07; Fig. 2). Remarkably, disease progression was retarded in patients with NSCLC treated with ICIs when compared with patients treated with SOC ([HR = 0.74; 95% CI, 0.67–0.80], Fig. 3), risk of death ([HR = 0.74; 95% CI, 0.70–0.79], Fig. 2), and increased ORR ([RR = 1.28; 95% CI, 1.18–1.39], Fig. 4). However, the benefit of ICI therapy in patients with SCLC was only indicated by OS ([HR = 0.82; 95% CI, 0.75–0.90], Fig. 2).

### Safety

**Fig. 5:**
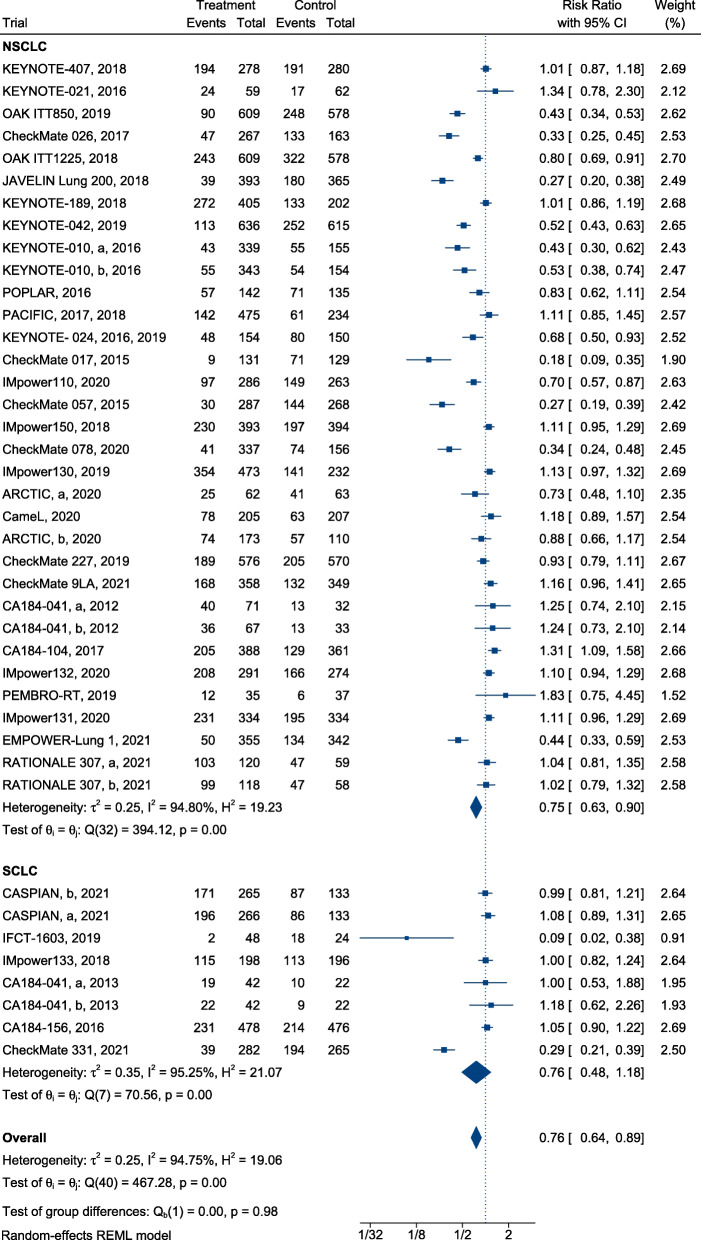
Forest plots of RRs comparing Grade 3–5 TRAEs of immunotherapy between NSCLC and SCLC

**Fig. 6:**
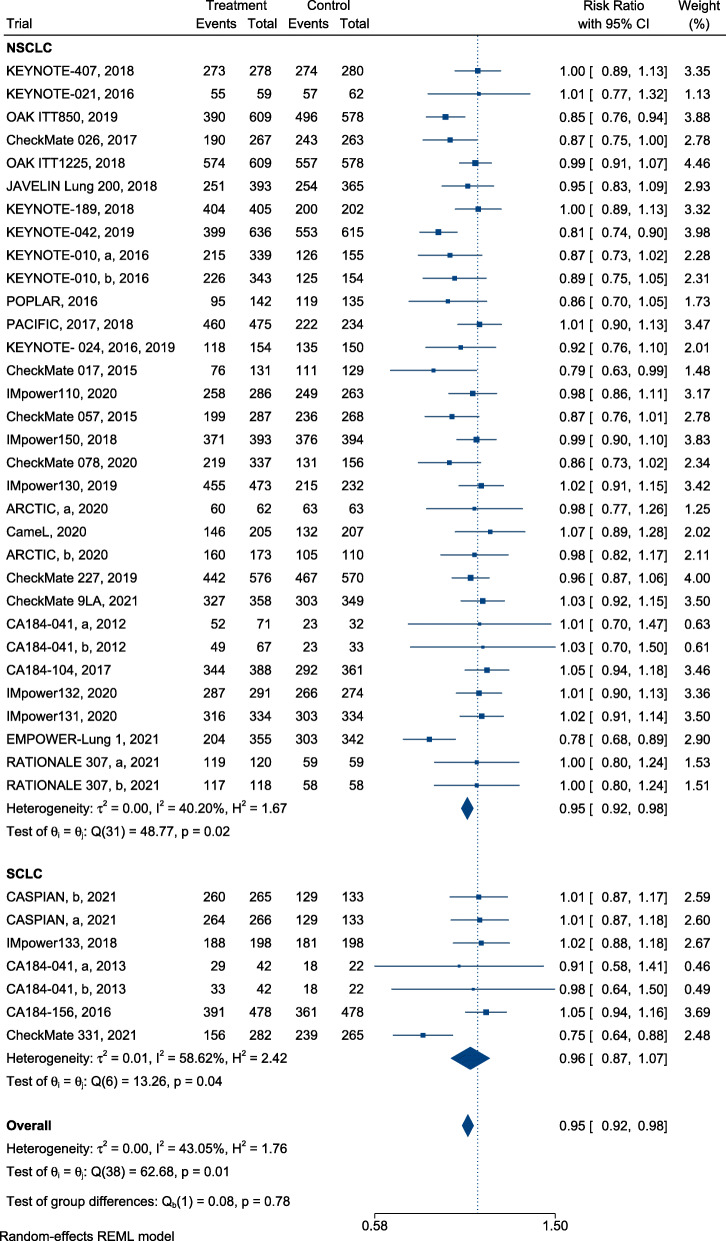
Forest plots of RRs comparing Grade 1–5 TRAEs of immunotherapy between NSCLC and SCLC

Compared with SOC alone, immunotherapy for patients with lung cancer reduced the risk of Grade 3–5 TRAEs (RR, 0.76; 95% CI, 0.64–0.89, Fig. 5) and Grade 1–5 TRAEs (RR, 0.95; 95% CI, 0.92–0.98, Fig. 6). In terms of Grade 3–5 TRAEs, no significant difference in risk reduction was observed among patients with different subtypes of lung cancer receiving ICI treatment when compared with SOC ([RR = 0.75; 95%, CI, 0.63–0.90] and [RR = 0.76; 95% CI, 0.48–1.18], respectively; difference *p* = 0.98; Fig. 5). The risk of Grade 1–5 TRAEs was equivalent among patients with different subtypes of lung cancer treated with ICIs and SOC ([RR = 0.95; 95% CI, 0.92–0.98] and [RR = 0.96; 95% CI, 0.87–1.07], respectively; *p* = 0.78; Fig. 6).

### Subgroup analysis

Table 2 and Table S3 display differences in the efficiency of ICI therapy between patients with NSCLC and SCLC. Importantly, as indicated by PFS, patients with NSCLC presented greater benefits following ICI therapy plus SOC than those with SCLC, when corresponding ICI-treated patients were used as a standard for comparison ([HR, 0.63; 95% CI, 0.57–0.69] and [HR, 0.83; 95% CI, 0.76–0.90], respectively, difference *p* < 0.01); similar results were observed following ICI monotherapy ([HR, 0.82; 95% CI, 0.73–0.91] and [HR, 1.68; 95% CI, 0.90–2.45], respectively; *p* = 0.03). Moreover, we further assessed differences on efficiency between patients with NSCLC and SCLC when immunotherapy was employed as the first or subsequent line of treatment. We detected an advantage in terms of PFS in patients with NSCLC when compared with patients with SCLC in both the first ([HR, 0.68; 95% CI, 0.60–0.76] and [HR, 0.83; 95% CI, 0.76–0.90], respectively, difference *p* = 0.01) and subsequent line of therapy ([HR, 0.83; 95% CI, 0.73–0.92] and [HR, 1.68; 95% CI, 0.90–2.45], respectively, *p =* 0.03). However, further subgroup analyses of sex, age, drug target, and Eastern Cooperative Oncology Group Performance Status (ECOG PS) score showed no statistically significant differences on PFS between patients with NSCLC and SCLC. In addition, we conducted subgroup analyses, including sex, age, smoking status, line of therapy, research methodology, drug target, and ECOG PS score, for OS and found no statistically significant differences in OS among patients with NSCLC and SCLC in all subgroups (Table S3).

**Table 2:** Differences in PFS benefits of Immunotherapy in NSCLC and SCLC by subgroups

Variable	Study	Test for Difference			
		NSCLC	SCLC	χ^**2**^	***P*** Value
**Overall**	41	0.74 [0.67; 0.80]	0.95 [0.77; 1.13]	5.03	0.02
**Sex**					
Male	18	0.63 [0.56; 0.69]	0.87 [0.64; 1.10]	3.16	0.08
Female	18	0.69 [0.57; 0.82]	0.59 [0.37; 0.81]	0.67	0.41
**Age**					
< 65 yr	18	0.62 [0.54; 0.70]	0.76 [0.54; 0.98]	1.40	0.24
≥ 65 yr	14	0.67 [0.58; 0.77]	0.76 [0.53; 0.99]	0.44	0.51
**Line of therapy**					
First	26	0.68 [0.60; 0.76]	0.83 [0.76; 0.90]	7.68	0.01
Subsequent	15	0.83 [0.73; 0.92]	1.68 [0.90; 2.45]	4.59	0.03
**Research methodology**					
ICI vs non-ICI	20	0.82 [0.73; 0.91]	1.68 [0.90; 2.45]	4.66	0.03
ICI + non-ICI vs non-ICI	21	0.63 [0.57; 0.69]	0.83 [0.76; 0.90]	15.28	< 0.01
**Drug target**					
Anti-PD-1/PD-L1	31	0.73 [0.56; 0.81]	1.16 [0.64; 1.68]	2.61	0.11
Anti-CTLA-4	6	0.84 [0.73; 0.95]	0.86 [0.76; 0.96]	0.07	0.80
Anti-PD-1/PD-L1 + CTLA-4	4	0.75 [0.66; 0.83]	0.84 [0.68; 0.99]	1.12	0.29
**ECOG PS**					
0	17	0.64 [0.54; 0.75]	0.84 [0.53; 1.15]	1.42	0.23
1	17	0.64 [0.57; 0.71]	0.72 [0.53; 0.92]	0.54	0.46
**Trial phase**					
II	8	0.75 [0.59; 0.91]	1.23 [0.54; 1.91]	1.17	0.18
III	33	0.73 [0.66; 0.81]	0.92 [0.71; 1.13]	2.65	0.10

## Discussion

The present study is the first systematic review and meta-analysis to evaluate the association between ICIs and long-term outcomes in patients with NSCLC and SCLC. We used published data from 38 RCTs of high quality, including more than 20,000 patients with lung cancer, revealing that ICIs were associated with a better therapeutic effect on reducing the risk of death in patients with NSCLC and SCLC without increasing TRAEs when compared with SOC. However, in terms of ORR and control of disease progression, benefits were primarily observed in patients with NSCLC, who showed significant improvements when compared with patients with SCLC. Compared with SOC, immunotherapy resulted in significantly prolonged PFS in patients with NSCLC than in patients with SCLC, with a significant difference noted between the two subgroups. Furthermore, among the treatment strategies, ICIs plus SOC led to a better improvement in PFS than ICI monotherapy in both patients with NSCLC and SCLC patients; accordingly, it is recommended for patients with advanced lung cancer as a preferential option. However, I^2^ > 50% in PFS analyses of NSCLC and SCLC indicated heterogeneity. In terms of NSCLC, we conducted subgroup analysis for drug targets, revealing that I^2^ of CTLA-4 and PD-1/PD-L1 plus CTLA-4 groups was 0 and 10.17% after grouping; however, the heterogeneity for the PD-1/PD-L1 group persisted (Fig. S2). On carefully comparing therapeutic regimens, we observed that the CTLA-4 and PD-1/PD-L1 plus CTLA-4 groups adopted similar ICI regimens among different trials. Nevertheless, the number of ICIs in the PD-1/PD-L1 group reached eight, with some employed in only one trial. Therefore, we believe that variations in ICIs possibly accounted for the heterogeneity. For SCLC, we found only two trials that assessed non-first-line treatment. Accordingly, we conducted a subgroup analysis for the line of therapy and observed that the I^2^ for first-line treatment became 0; this suggested that the different non-first-line treatments were sources of heterogeneity (Fig. S3).

Furthermore, the current study indicated that the magnitude of immunotherapy treatment effects was related to the ICI drug targets. Based on the checkpoints, ICIs are roughly classified as anti-PD-1/ PD-L1 and anti-CTLA-4 drugs. Some researchers have highlighted that combining anti-PD-1/ PD-L1 with anti-CTLA-4 might lead to additive antitumour effects [16]. Herein, we demonstrated that, among different drug targets, the combination of anti-PD-1/PD-L1 and anti-CTLA-4 decreased the risk of death by 28% in patients with NSCLC, which was only 26% in the anti-PD-1/PD-L1 group and 9% in the anti-CTLA-4 group, consistent with the former hypothesis. Similarly, the magnitude of PFS benefits seemed to favour anti-PD-1/PD-L1 plus anti-CTLA-4 treatment in both patients with NSCLC and SCLC. Nevertheless, the magnitude of OS benefits favoured the anti-PD-1/PD-L1 group most in patients with SCLC, revealing that the combination of anti-PD-1/PD-L1 and anti-CTLA-4 treatment has better therapeutic effects in patients with NSCLC. Given the limited number of clinical trials, additional research is needed to comprehensively evaluate the efficiency of drug combinations. Nevertheless, there is a potential explanation for the promising effects of combined anti-PD-1/PD-L1 with anti-CTLA-4 treatment. Although the anti-PD-1/PD-L1 and anti-CTLA-4 antibodies are distinct ICIs, they may play a synergistic role. More precisely, anti-PD-1/PD-L1 antibodies restore the antitumour function of T cells, whereas anti-CTLA-4 antibodies activate antitumour T-cell responses and induce the proliferation of T-cells involving memory T cells [49].

In addition, we observed that therapy with ICIs plus SOC conferred greater treatment benefits than ICI monotherapy. This finding was in line with findings reported by Wang and colleagues, which revealed that ICI plus SOC results in significantly prolonged PFS when compared with monotherapy with immunotherapy [50]. However, we compared both NSCLC and SCLC rather than just NSCLC. In theory, chemotherapy or radiotherapy can induce the expression of immune checkpoints on infiltrating immune cells and tumour cells, which might enhance the curative effects of ICI therapy [50]. Thus, a combination of ICIs and SOCs should be adopted as the optimal treatment for SCLC and NSCLC. For NSCLC, we recommended a combination of SOC and anti-PD-1/PD-L1 plus anti-CTLA-4 antibodies. Furthermore, although men and women exhibited distinct immunological responses to antigens, no significant association of sex in terms of survival and disease control advantages was detected in patients with NSCLC and SCLC, in agreement with a previous study by Wallis et al. [51].

Currently, no RCTs comparing the therapeutic effects of ICIs in patients with NSCLC and SCLC patients have been reported. In the past decade, most drugs were found to be ineffective in SCLC management, in contrast to the success in the NSCLC field. In 2018, the IMpower133 trial revealed that the combination of atezolizumab and chemotherapy significantly prolonged OS and PFS when compared with chemotherapy alone for patients with advanced-stage SCLC [19]; this challenged the traditional chemotherapy-based treatment strategies for patients with SCLC. Subsequently, atezolizumab was adopted as the first-line treatment for SCLC. To date, only one study has compared first-line treatment strategies for SCLC, which only included two studies of ICI therapy, while most other trials in the SCLC field were limited in chemotherapy subtypes [52]. Another novelty of our study lies in the subgroup analyses according to individual conditions and treatment methods. Herein, we demonstrated that for patients with SCLC, ICI plus SOC therapy confers superior advantages over SOC, as indicated by OS and PFS. Furthermore, our study revealed that patients with NSCLC presented greater PFS benefits than SCLC patients receiving ICI monotherapy and ICI plus SOC therapy regarding different lung cancer subtypes. In terms of the line of therapy, patients with NSCLC benefited more from ICI treatment than patients with SCLC in both the first and subsequent lines of therapy, with significant differences between groups. These findings indicate that NSCLC might benefit more from ICI treatment than SCLC, regardless of the methodology of drug administration.

### Implications of the study

#### Providing optimal treatment strategies for patients with lung cancer

Our study had several clinical implications. We recommend treatment strategies for patients with lung cancer based on sufficient evidence. With the development and gradual maturity of ICI treatment, it is necessary for oncologists, respiratory physicians, and thoracic surgeons to navigate multiple treatment strategies, including various ICI therapies, and to determine the optimal treatment for patients with lung cancer. Therefore, we recommend that patients with SCLC undergo ICI plus SOC therapy based on findings in the present study. For patients with NSCLC, a combination of anti-PD-1/PD-L1 and anti-CTLA-4 antibodies and SOC could serve as the optimal treatment strategy.

#### Discovering novel therapeutic regimen for SCLC

In addition, our research provides a new approach for SCLC therapy. The median OS for SCLC, especially for extensive-stage SCLC, is less than 10 months, emphasising the need for novel promising treatments [2]. However, several clinical trials, including targeted drugs, have declared treatment failure for SCLC in the past few decades. In 2013 and 2016, CA184–041 and CA184–156 were conducted by Reck et al. to evaluate the therapeutic effect of ipilimumab in patients with SCLC patients. The authors reported that ipilimumab had no significant efficacy when compared with traditional chemotherapy [20, 21]. Recently, IMpower133 and CASPIAN assessed anti-PD-1/PD-L1 antibodies with or without anti-CTLA-4 antibodies as the first-line of therapy for patients with SCLC, revealing better therapeutic effects in prolonging OS and PFS in patients with SCLC than chemotherapy [16, 19], which indicated a major development in SCLC therapy. However, IFCT-1603 and CheckMate 331 used anti-PD-1/PD-L1 antibodies as first-line therapy when compared with traditional chemotherapy and observed no significant difference in prolonging OS. In terms of PFS, immunotherapy led to worse results than chemotherapy [17, 18]. In the current study, we systematically analysed data from these RCTs and validated that ICI therapy could prolong OS in patients with SCLC. Considering these discrepancies, we conducted subgroup analyses in line with therapy and drug targets, which recommended ICI treatment as the first-line therapy for SCLC, affording better OS and PFS than with the subsequent line of therapy. Among different drug targets, anti-PD-1/PD-L1 antibodies with or without anti-CTLA-4 antibodies presented a superior advantage in reducing the risk of death; this indicated that anti-PD-1/PD-L1 antibodies with or without anti-CTLA-4 antibodies should be adopted as the first-line therapy for patients with SCLC. Moreover, additional trials should be conducted to further validate the treatment effects of anti-PD-1/PD-L1 antibodies with or without anti-CTLA-4 antibodies as the first-line therapy for SCLC.

#### Landscape of ICI treatment efficacy among lung cancer

Another clinical implication of our study is that NSCLC might benefit more from ICI therapy than SCLC among different histological subtypes. Currently, available studies are insufficient to compare the treatment effects of ICIs in patients with NSCLC and SCLC. However, we conducted the first analysis to evaluate differences in ICI treatment between patients with NSCLC and SCLC. The results revealed that patients with NSCLC benefited more from immunotherapy than patients with SCLC in almost all subgroups, regardless of treatment methodology and individual patient conditions. Notably, ICI treatment presented a statistically significant advantage in terms of therapeutic efficiency in patients with NSCLC when compared with patients with SCLC, irrespective of first or subsequent line of therapy and treatment methodology (ICIs alone or ICIs plus SOC). In terms of PFS and ORR, patients with SCLC receiving immunotherapy showed no difference from those on SOC regimens, both of which were significantly lower than in patients with NSCLC. Thus, the above results demonstrated that although the OS of patients with SCLC could benefit from immunotherapy, PFS and ORR fail to demonstrate promising effects equivalent to those in patients with NSCLC.

#### Strengthens and weaknesses of this study

First, this is the first study to comprehensively review the relative benefits and risks of ICI treatment between patients with NSCLC and SCLC and indirectly compare the efficiency of treatment methodology in each histological lung cancer subtype, including the largest number of trials and patients. As few studies have analysed the efficiency and safety of ICI treatment in patients with SCLC, and no comparison directly included patients with SCLC versus those with NSCLC, to a certain extent, we bridged the gap in efficiency and safety data for ICI therapy among patients with NSCLC and SCLC. Previously, Maung et al. have shown that ICIs conferred better survival benefits than chemotherapy in both NSCLC and SCLC [53]. However, their conclusions were mainly based on qualitative analysis, without data analysis of clinical trials. In contrast, the quantitative analysis in our study could lead to more accurate and convincing results. Furthermore, our findings confirmed that immunotherapy could better benefit patients with NSCLC in prolonging PFS and increasing ORR than patients with SCLC. Given that the therapeutic effects of ICI treatment for SCLC remain controversial, we conducted a comprehensive assessment to compare its efficacy with chemotherapy. We observed that ICIs could undoubtedly reduce the risk of death in patients with SCLC, with a statistically significant difference, which has compensated for the lack of assessments of immunotherapy in the SCLC field. Second, one of the distinct strengths of our study is the data quality involved in our analyses. We employed 38 well-designed RCTs, gathered data from more than 20,000 patients with lung cancer, and carried out analyses according to predefined primary endpoints of OS and PFS and second endpoints of TRAEs with different grades. Our study was the largest scale of ICI analyses in patients with lung cancer. Under most circumstances, one essential factor in reducing statistical errors in a meta-analysis involves a large-scale quantity of subjects with high quality. Third, this study recommends optimal ICI treatment strategies in patients with NSCLC and SCLC. For NSCLC, the combination of anti-PD-1/PD-L1 and anti-CTLA-4 antibodies plus SOC is recommended for both first and subsequent lines of immunotherapy. In patients with SCLC, we only recommend the first-line treatment as anti-PD-1/PD-L1 plus SOC with or without anti-CTLA-4 antibodies.

Despite these strengths, several limitations exist in the present study. First, differences in risks and benefits between patients with NSCLC and SCLC were determined and compared through indirect analyses. To date, no RCTs have directly compared the efficiency and safety of immunotherapy between patients with SCLC and patients with NSCLC. Therefore, our results remain suggestive but not conclusive. Second, although our study is based on the largest scale of ICI analysis for lung cancer, more research is needed to comprehensively investigate the efficiency of immunotherapy in SCLC. Third, in selecting immunotherapy, the risk of toxicity is as important as the therapeutic effect, which should be thoroughly investigated. However, we only considered TRAEs of grade ≥ 3 and any grade, as information regarding TRAEs stratified by predefined subgroups was unavailable. Furthermore, additional factors should be used to evaluate toxicity.

## Conclusion

In conclusion, for patients with NSCLC and SCLC, ICI therapies are promising therapeutic options with advantages in terms of survival and toxicity over SOC. Furthermore, ICIs plus SOC are recommended as the optimal first-line therapy for patients with SCLC. Anti-PD-1/PD-L1 plus SOC with anti-CTLA-4 antibodies is recommended for patients with NSCLC without mutated gene targets in both the first and subsequent lines of therapy. In addition, immunotherapy as a subsequent line is not recommended as a standard strategy for patients with SCLC.

## Supplementary Information

**Additional file 1: Fig. S1** Funnel plot of the effect size for each trial. **Fig. S2** Drug targets analysis for NSCLC. **Fig. S3** Therapeutic scheme analysis for SCLC. **Table S1** Search strategies. **Table S2** The methodological quality of included RCTs. **Table S3** Differences in OS benefits of Immunotherapy in NSCLC and SCLC by subgroups

## Data Availability

All data generated or analyzed during this study are included in this published article [and its supplementary information files]. All the data were available from the corresponding authors for reasonable request.

## Abbreviations

ATE: Atezolizumab
CI: Confidence interval
CTLA-4: Cytotoxic T lymphocyte-associated antigen 4
DOC: Docetaxel
ECOG PS: Eastern Cooperative Oncology Group Performance Status
HR: Hazard ratio
ICI: Immune checkpoint inhibitor
IPI: Ipilimumab
NIV: Nivolumab
DUR: Durvalumab
TRE: Tremelimumab
CAM: Camrelizumab
CEM: Cemiplimab
TIS: Tislelizumab
SCLC: Small-cell lung cancer
NSCLC: Non-small-cell lung cancer
ORR: Objective response rate
OS: Overall survival
PBC: Platinum-based chemotherapy
PD-1: Programmed cell death 1
PD-L1: Programmed cell death 1 ligand 1
PEM: Pembrolizumab
PFS: Progression-free survival
RCT: Randomised controlled trial
RR: Risk ratio
TRAEs: Treatment-related adverse events
SOC: Standard of care
