# Supplementary material for: The landscape of immune checkpoint inhibitor therapy in advanced lung cancer
Source: BMC Cancer. 2021 Aug 28;21:968. doi: 10.1186/s12885-021-08662-2 (PMC8403352; doi:10.1186/s12885-021-08662-2)

**Table S1. Search strategies (20, 699results)**

**PubMed**: 14, 692 results

((Toripalimab[Mesh] OR “Cell Death 1 Inhibitor”[tw] OR “drug therapy*”[tw]) OR (Sintilimab[Mesh] OR “Antibodies”[tw] OR “IBI308”[tw]) OR (Camrelizumab[Mesh] OR “drug therapy*”[tw] OR “SHR 1210”[tw]) OR (Tislelizumab[Mesh] OR “therapeutic use*”[tw] OR “Antibodies”[tw]) OR (Nivolumab[Mesh] OR “therapeutic use*”[tw] OR Opdivo[tw] OR ONO-4538[tw] OR BMS-936558[tw] OR Nivo[tw] OR Pembrolizumab[tw] OR keytruda[tw] OR “SCH 900475”[tw] OR MK-3475[tw]) OR (avelumab[Mesh] OR Atezolizumab[tw] OR MSB0010718C[tw] OR Tecentriq[tw] OR RO5541267[tw] OR RG7446[tw] OR MPDL3280A[tw]) OR (Durvalumab[Mesh] OR MEDI-4736[tw] OR MEDI4736[tw]) OR (Ipilimumab[Mesh] OR Yervoy[tw] OR MDX-CTLA-4[tw]) OR (Tremelimumab[Mesh] OR “Drug therapy*”[tw]) OR (“Programmed Cell Death 1 Receptor”[Mesh] OR “B7-H1 Antigen”[Mesh] OR PD-1[tw] OR PD-L1[tw] OR “cytotoxic T-lymphocyte associated antigen-4”[tw] OR CTLA-4[tw] OR “immune checkpoint inhibitor”[tw])) AND (“Carcinoma, Non-Small-Cell Lung”[Mesh] OR SCLC[tw] OR “lung carcinoma”[tw] OR “pulmonary carcinoma”[tw] OR “lung cancer”[tw] OR “lung tumor”[tw]) AND (“Randomized Controlled Trials as Topic”[Mesh] OR Trail[tw] OR clinical trials as topic[tw] OR study[tw] OR “randomized controlled trial”[tw])

**Embase:**6,485 results

((sintilimab OR toripalimab OR ibi308 OR camrelizumab OR (shr AND 1210) OR tislelizumab OR nivolumab OR opdivo OR 'ono 4538' OR 'mdx 1106' OR 'bms 936558' OR nivo OR pembrolizumab OR lambrolizumab OR keytruda OR sch 900475 OR 'mk 3475' OR atezolizumab OR tecentriq OR durvalumab OR 'medi 4736' OR medi4736 OR avelumab OR ipilimumab OR 'mdx ctla 4' OR yervoy OR tremelimumab OR immiune checkpoint inhibitor OR 'programmed cell death 1' OR 'pd 1' OR 'programmed cell death ligand 1' OR 'pd l1' OR 'cytotoxic t lymphocyte associated antigen 4' OR 'ctla 4') AND （lung cancer OR lung tumor OR nsclc OR sclc OR lung carcinoma) AND 'randomized controlled trial'

**Cochrane**: 8, 467 results

('sintilimab' OR 'toripalimab' OR 'IBI308' OR 'camrelizumab' OR 'SHR 1210' OR 'tislelizumab'OR'Pembrolizumab' OR 'lambrolizumab' OR 'keytruda' OR 'SCH 900475' OR 'MK- 3475' OR 'Nivolumab' OR 'Opdivo' OR 'ONO-4538' OR 'MDX-1106' OR 'BMS-936558' OR 'Nivo' OR ' Atezolizumab' OR 'MSB0010718C' OR 'Tecentriq' OR 'RO5541267' OR 'RG7446' OR 'MPDL3280A' OR 'Durvalumab' OR 'MEDI-4736' OR 'MEDI4736' OR 'Avelumab' OR ' Ipilimumab ' OR 'Tremelimumab' OR 'checkpoint inhibitor' OR 'programmed cell death-1' OR 'PD-1' OR ' programmed cell death ligand-1' OR 'PD-L1' OR 'cytotoxic T-lymphocyte associated antigen-4' OR 'CTLA-4') AND ('lung carcinoma' OR 'pulmonary carcinoma' OR 'lung cancer' OR 'lung tumor' OR 'NSCLC' OR 'SCLC') AND ( 'trials')

**Medicine**: 640 results

('sintilimab' OR 'toripalimab' OR 'IBI308' OR 'camrelizumab' OR 'SHR 1210' OR 'tislelizumab'OR'Pembrolizumab' OR 'lambrolizumab' OR 'keytruda' OR 'SCH 900475' OR 'MK- 3475' OR 'Nivolumab' OR 'Opdivo' OR 'ONO-4538' OR 'MDX-1106' OR 'BMS-936558' OR 'Nivo' OR ' Atezolizumab' OR 'MSB0010718C' OR 'Tecentriq' OR 'RO5541267' OR 'RG7446' OR 'MPDL3280A' OR 'Durvalumab' OR 'MEDI-4736' OR 'MEDI4736' OR 'Avelumab' OR ' Ipilimumab ' OR 'Tremelimumab' OR 'checkpoint inhibitor' OR 'programmed cell death-1' OR 'PD-1' OR ' programmed cell death ligand-1' OR 'PD-L1' OR 'cytotoxic T-lymphocyte associated antigen-4' OR 'CTLA-4') AND ('lung carcinoma' OR 'pulmonary carcinoma' OR 'lung cancer' OR 'lung tumor' OR 'NSCLC' OR 'SCLC') AND （'randomized controlled trials' OR 'randomized controlled test'）

**Table S2. The methodological quality of included RCTs**

| Study | | Year | A | B | C | D | E | F | Total |  |
| --- | --- | --- | --- | --- | --- | --- | --- | --- | --- | --- |
| CheckMate 017 | | 2015 | √ |  |  | √ | √ | √ | 4 |  |
| CheckMate 026 | | 2017 | √ |  |  | √ | √ | √ | 4 |  |
| CheckMate 057 | | 2015 | √ |  |  | √ | √ | √ | 4 |  |
| CheckMate 078 | | 2019 | √ |  |  | √ | √ | √ | 4 |  |
| JAVELIN Lung 200 | | 2018 | √ |  |  | √ | √ | √ | 4 |  |
| KEYNOTE-010(a) | | 2016 | √ | √ |  | √ | √ | √ | 5 |  |
| KEYNOTE-010(b) | | 2016 | √ | √ |  | √ | √ | √ | 5 |  |
| KEYNOTE-042 | | 2019 | √ |  |  | √ | √ | √ | 4 |  |
| OAK ITT850 | | 2017, 2019 | √ |  |  | √ | √ | √ | 4 |  |
| OAK ITT1225 | | 2018 | √ |  |  | √ | √ | √ | 4 |  |
| POPLAR | | 2016 | √ |  |  | √ | √ | √ | 4 |  |
| CA184-041, a | | 2012 | √ |  | √ | √ | √ | √ | 5 |  |
| CA184-041, b | | 2012 | √ |  | √ | √ | √ | √ | 5 |  |
| CA184-104 | | 2017 | √ |  | √ | √ | √ | √ | 5 |  |
| CheckMate 227 | | 2019 | √ | √ | √ | √ | √ | √ | 6 |  |
| IMpower130 | | 2019 | √ | √ | √ | √ | √ | √ | 6 |  |
| IMpower131 | | 2020 | √ | √ |  | √ | √ | √ | 5 |  |
| IMpower132 | | 2020 | √ | √ |  | √ | √ | √ | 5 |  |
| IMpower150 | | 2018 | √ | √ | √ | √ | √ | √ | 6 |  |
| KEYNOTE-021 | | 2016 | √ | √ | √ | √ | √ | √ | 6 |  |
| KEYNOTE-189 | | 2018 | √ | √ | √ | √ | √ | √ | 6 |  |
| KEYNOTE-407 | | 2018 | √ | √ | √ | √ | √ | √ | 6 |  |
| PACIFIC | | 2017, 2018 | √ |  | √ | √ | √ | √ | 5 |  |
| KEYNOTE- 024 | | 2016, 2019 | √ | √ |  | √ | √ | √ | 5 |  |
| IMpower110 | | 2020 | √ |  |  | √ | √ | √ | 4 |  |
| ARCTIC (a) | | 2020 | √ |  |  | √ | √ | √ | 4 |  |
| ARCTIC (a) | | 2020 | √ |  |  | √ | √ | √ | 4 |  |
| CameL | | 2020 | √ | √ |  | √ | √ | √ | 5 |  |
| CheckMate 9LA | | 2021 | √ | √ |  | √ | √ | √ | 5 |  |
| PEMBRO-RT | | 2019 | √ |  |  | √ | √ | √ | 4 |  |
| CASPIAN, a | | 2021 | √ | √ |  | √ | √ | √ | 5 |  |
| CASPIAN, b | | 2021 | √ | √ |  | √ | √ | √ | 5 |  |
| IFCT-1603 | | 2019 | √ |  |  | √ | √ | √ | 4 |  |
| IMpower133 | | 2018 | √ |  | √ | √ | √ | √ | 5 |  |
| CA184-041, a | | 2013 | √ |  | √ | √ | √ | √ | 5 |  |
| CA184-041, b | | 2013 | √ |  | √ | √ | √ | √ | 5 |  |
| CA184-156 | | 2016 | √ |  | √ | √ | √ | √ | 5 |  |
| CheckMate 331 | | 2021 | √ | √ |  | √ | √ | √ | 5 |  |
| EMPOWER-Lung 1 | | 2021 | √ |  |  | √ | √ | √ | 4 |  |
| RATIONALE 307(a) | | 2021 | √ | √ |  | √ | √ | √ | 5 |  |
| RATIONALE 307(b) | | 2021 | √ | √ |  | √ | √ | √ | 5 |  |
|  | A: Sequence generation; B: Allocation concealment; C: Blinding of participants, personnel and outcome assessors; D: Incomplete outcome data; E: No selective outcome reporting; F: Other sources of bias; √: low risk | | | | | | | | | |

**Table S3. Differences in OS benefits of Immunotherapy in NSCLC and SCLC by subgroups.**

| **Variable** | **Study** |  | **Test for Difference** | |  |  |  |
| --- | --- | --- | --- | --- | --- | --- | --- |
|  |  | **NSCLC** | | **SCLC** | | **χ^2^** | **P Value** |
| **Overall** | 39 | 0.74 [0.70; 0.79] | | 0.82 [0.75; 0.90] | | 3.22 | 0.07 |
| **Sex** |  |  | |  | |  |  |
| Male | 26 | 0.74 [0.68; 0.80] | | 0.85 [0.74; 0.96] | | 2.97 | 0.09 |
| Female | 26 | 0.72 [0.61; 0.82] | | 0.82 [0.65; 0.99] | | 1.04 | 0.31 |
| **Age** |  |  | |  | |  |  |
| < 65 yr | 25 | 0.71 [0.64; 0.78] | | 0.86 [0.73; 1.00] | | 3.66 | 0.06 |
| ≥65 yr | 17 | 0.75 [0.67; 0.82] | | 0.78 [0.59; 0.96] | | 0.09 | 0.77 |
| **Smoking status** |  |  | |  | |  |  |
| Nonsmoker | 15 | 0.79 [0.59; 1.00] | | 0.55 [0.19; 0.91] | | 1.31 | 0.25 |
| Ever smoker | 16 | 0.73 [0.67; 0.79] | | 0.80 [0.69; 0.91] | | 0.94 | 0.33 |
| **Line of therapy** |  |  | |  | |  |  |
| First | 24 | 0.76 [0.69; 0.83] | | 0.81 [0.72; 0.91] | | 0.89 | 0.35 |
| Subsequent | 15 | 0.73 [0.68; 0.78] | | 0.86 [0.70; 1.01] | | 2.44 | 0.12 |
| **Research methodology** |  |  | |  | |  |  |
| ICI vs non-ICI | 20 | 0.74 [0.69; 0.79] | | 0.86 [0.70; 1.01] | | 2.11 | 0.15 |
| ICI + non-ICI vs non-ICI | 19 | 0.75 [0.67; 0.83] | | 0.81 [0.72; 0.91] | | 0.93 | 0.33 |
| **Drug target** |  |  | |  | |  |  |
| Anti-PD-1/PD-L1 | 29 | 0.73 [0.68; 0.78] | | 0.78 [0.68; 0.87] | | 0.70 | 0.40 |
| Anti-CTLA-4 | 6 | 0.91 [0.78; 1.04] | | 0.92 [0.79; 1.05] | | 0.01 | 0.94 |
| Anti-PD-1/PD-L1 + CTLA-4 | 4 | 0.72 [0.65; 0.80] | | 0.82 [0.66; 0.98] | | 1.14 | 0.29 |
| **ECOG PS** |  |  | |  | |  |  |
| 0 | 21 | 0.71 [0.63; 0.80] | | 0.97 [0.67; 1.27] | | 2.62 | 0.11 |
| 1 | 21 | 0.74 [0.67; 0.83] | | 0.86 [0.68; 1.03] | | 1.37 | 0.24 |
| **Trial phase** |  |  | |  | |  |  |
| II | 8 | 0.74 [0.60; 0.89] | | 0.83 [0.57; 1.10] | | 0.33 | 0.57 |
| III | 31 | 0.74 [0.70; 0.79] | | 0.82 [0.74; 0.90] | | 2.64 | 0.10 |

**Figure S1. Funnel plot of the effect size for each trial.**


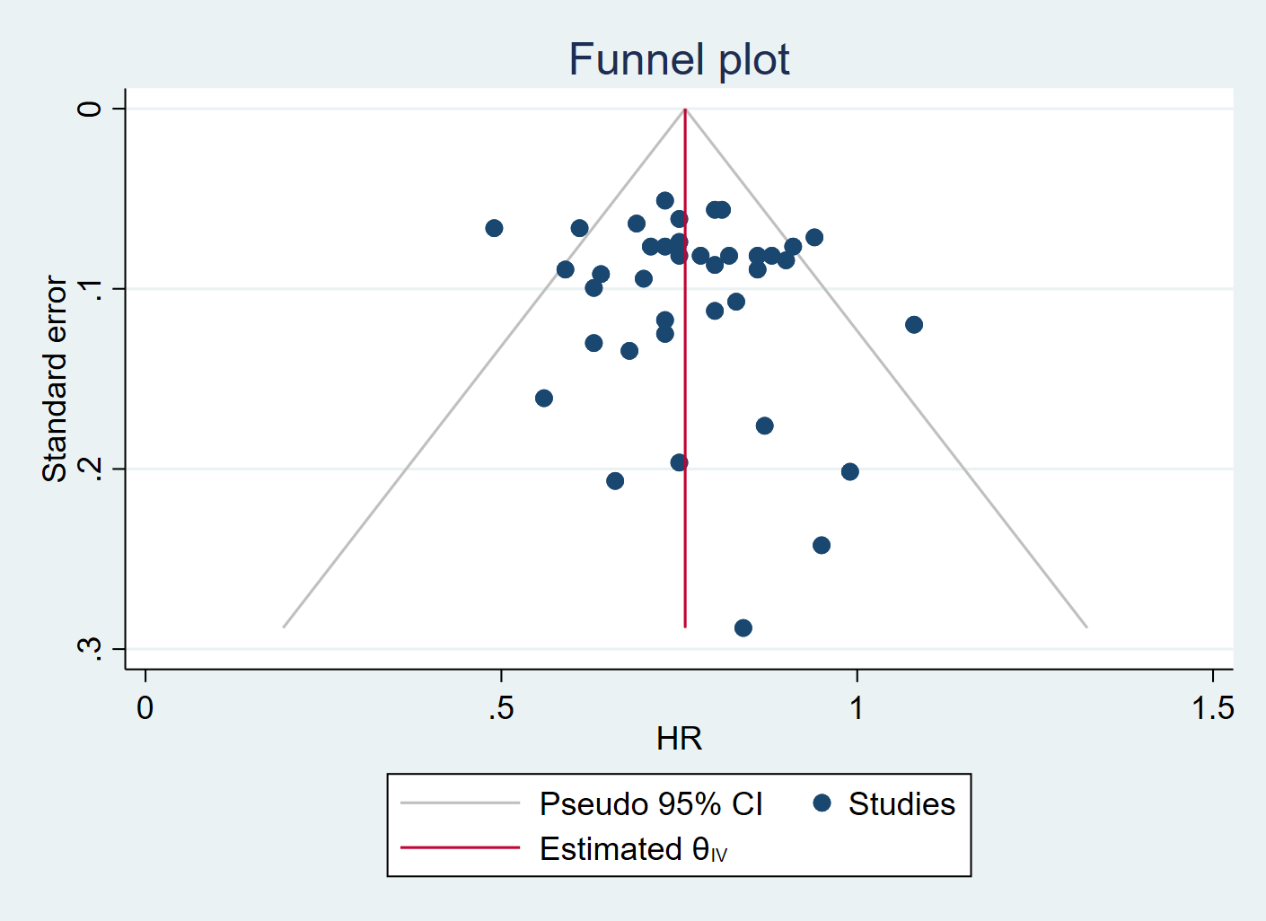


**Figure S2. Drug targets analysis for NSCLC**


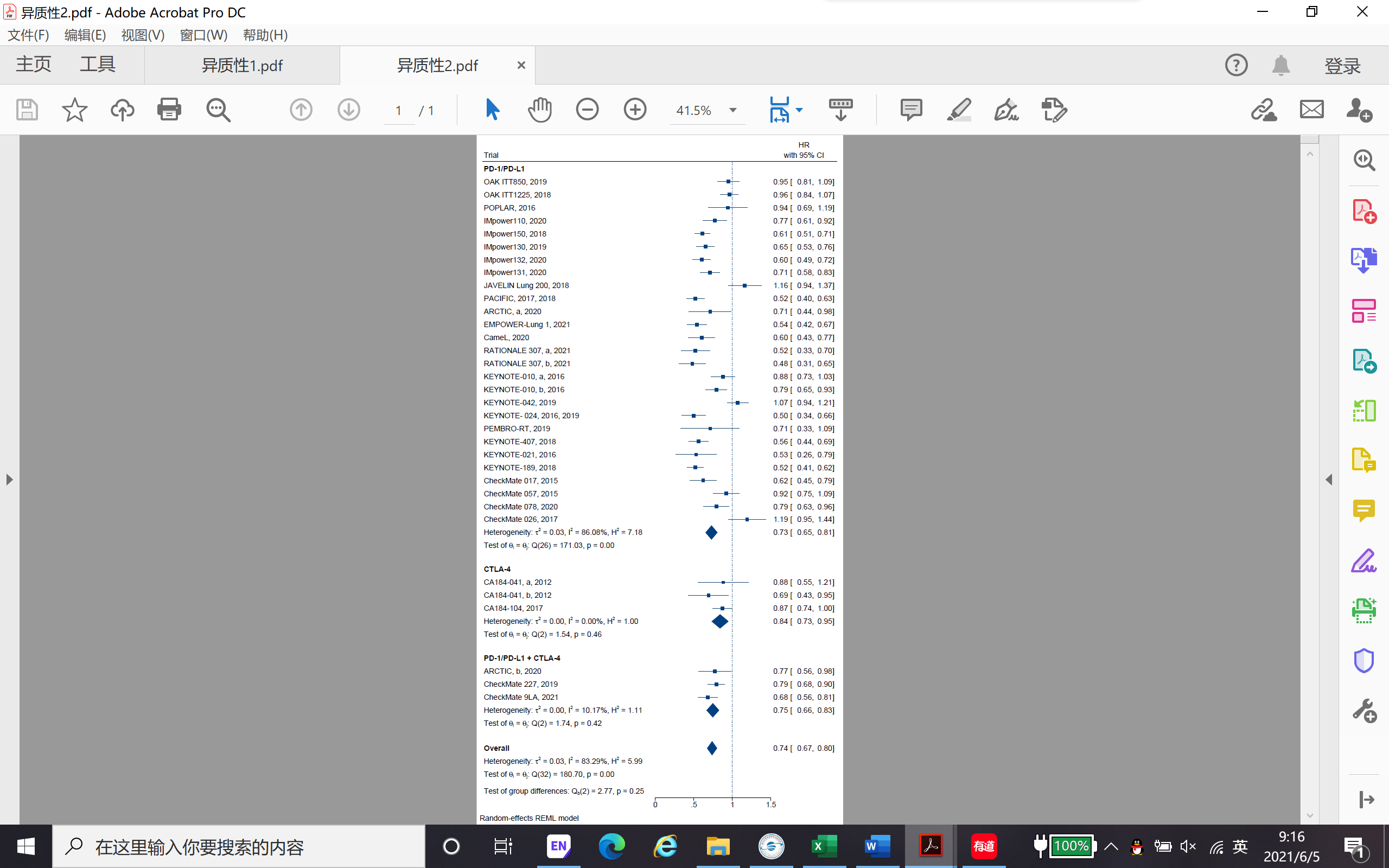


**Figure S3. Therapeutic scheme analysis for SCLC**


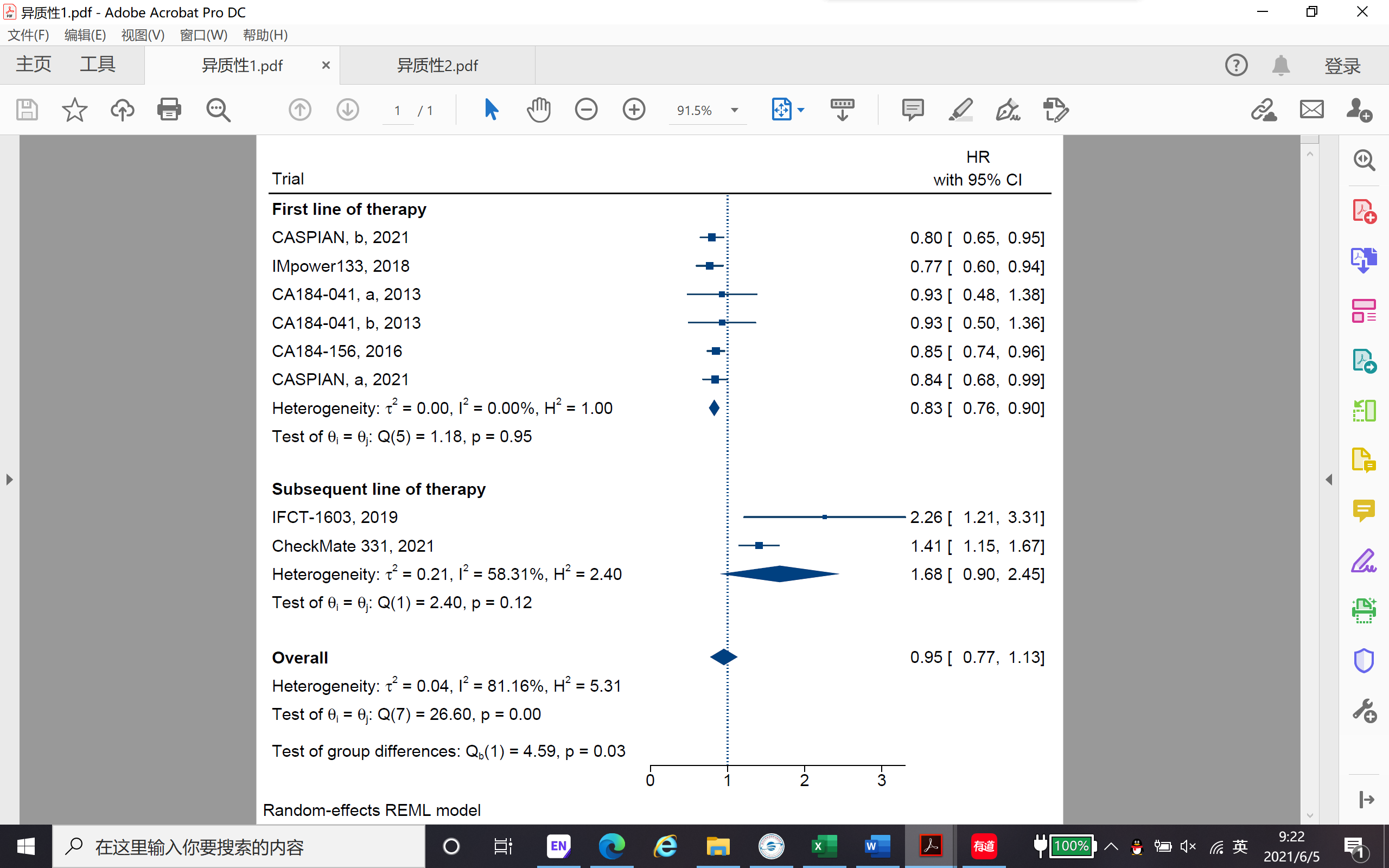

Supplement: Supplementary file 1 — Additional file 1: Fig. S1 Funnel plot of the effect size for each trial. Fig. S2 Drug targets analysis for NSCLC. Fig. S3 Therapeutic scheme analysis for SCLC. Table S1 Search strategies. Table S2 The methodological quality of included RCTs. Table S3 Differences in OS benefits of Immunotherapy in NSCLC and SCLC by subgroups [file 12885_2021_8662_MOESM1_ESM.docx]
